# Design and Generation of MLPA Probe Sets for Combined Copy Number and Small-Mutation Analysis of Human Genes: EGFR as an Example

**DOI:** 10.1100/tsw.2010.195

**Published:** 2010-10-12

**Authors:** Malgorzata Marcinkowska, Kwok-Kin Wong, David J. Kwiatkowski, Piotr Kozlowski

**Affiliations:** ^1^ Laboratory of Cancer Genetics, Institute of Bioorganic Chemistry, Polish Academy of Sciences, Poznan, Poland; ^2^ Department of Medical Oncology, Dana-Farber Cancer Institute, Boston, MA, USA; ^3^ Ludwig Center at Dana-Farber/Harvard Cancer Center, Boston, MA, USA; ^4^ Division of Translational Medicine, Department of Medicine, Brigham and Women's Hospital, Harvard Medical School, Boston, MA, USA

**Keywords:** multiplex ligation-dependent probe amplification, MLPA, copy number variation, CNV, EGFR, large deletion, amplification, mutation detection

## Abstract

Multiplex ligation-dependent probe amplification (MLPA) is a multiplex copy number analysis method that is routinely used to identify large mutations in many clinical and research labs. One of the most important drawbacks of the standard MLPA setup is a complicated, and therefore expensive, procedure of generating long MLPA probes. This drawback substantially limits the applicability of MLPA to those genomic regions for which ready-to-use commercial kits are available. Here we present a simple protocol for designing MLPA probe sets that are composed entirely of short oligonucleotide half-probes generated through chemical synthesis. As an example, we present the design and generation of an MLPA assay for parallel copy number and small-mutation analysis of the *EGFR* gene.

## INTRODUCTION

Copy number variation (CNV) in the human genome has become well recognized in recent years. CNVs are heritable and somatic losses and gains of DNA segments that range in size from <1 kb to >1 Mb, and may include entire genes or even multiple genes[1,2]. The physiological effects of CNVs are a subject of continuing investigation, and range from neutral to phenotype-modifying to disease-causing mutations. Polymorphic CNVs account for about 10% of the human genome, overlapping hundreds of genes. Genomic deletion mutations occurring in genes that cause Mendelian disorders are a special subcategory of germline CNVs, and account for up to 70% of all mutations seen in some genes (e.g., *BRCA1*, *DMD*, *TSC2*, *STK11*)[3,4,5,6,7]. In addition, it is well known that CNV is widespread throughout the typical cancer genome and very likely contributes to cancer pathogenesis as much as point mutations[2,8]. A

number of methods have been developed to assess CNV at the genome-wide level. Array comparative genomic hybridization, high-density single nucleotide polymorphism (SNP) arrays (reviewed in [9]), and, more recently, second-generation sequencing[10] are widely used for CNV identification, and major improvements (regarding the precision of CNV genotyping and breakpoint mapping) to these methods have recently been achieved[11,12,13]. However, the major laboratory tool for the analysis of CNV mutations over small genomic regions, particularly for clinical diagnostic laboratories, is multiplex ligation-dependent probe amplification (MLPA) (reviewed in [14,15]).

MLPA is a method first described by Schouten et al.[16] 8 years ago as a multiplex assay utilizing up to 45 probes specific for different genomic locations (often exons in a gene of interest). Each probe is composed of two sister half-probes (a 5’ half-probe and a 3’ half-probe). The first step of the MLPA procedure is hybridization, during which the sister half-probes hybridize to adjacent target sequences in the input genomic DNA. In the next step, ligation of sister half-probes is performed under stringent conditions, and then the ligation products are amplified by polymerase chain reaction (PCR) using fluorescently tagged universal primers to sequences incorporated in the sister half-probes (Fig. 1A). The PCR products are separated by capillary electrophoresis (CE) (Fig. 1C), and the signal from each probe is normalized against a control probe signal and is compared to a corresponding normalized signal observed in a set of reference samples (Fig. 1D).

Originally, MLPA was designed as a copy number analysis tool, and it has been successfully used in the testing and identification of hundreds of large mutations in numerous disease-related genes, including *DMD*, *BRCA1*, *NF1*, *STK11*, and *TSC2*. Further modifications of the MLPA protocol broadened its range of applications. The additional applications of MLPA are SNP genotyping[16], methylation status determination[17], copy number analysis in segmentally duplicated regions[18,19], expression profiling[20], mouse transgene genotyping[21], analysis of DNaseI hypersensitive sites[22], determination of the effectiveness of conditional allele conversion[23], and strand-specific expression analysis (Mykowska et al., submitted for publication).

The main disadvantage of the standard MLPA setup is a complicated and time-consuming (and therefore expensive) process of probe design and generation. This is due to the necessity for creating long 3’ half-probes (~100–400 nt). Usually this is done by cloning 3’ half-probes in specially prepared M13 vectors, enabling insertion of arbitrary numbers of nucleotides into those probes[16]. In practice, this disadvantage seriously limits the applicability of MLPA to novel genes or sets of genes for which ready-to-use commercial kits are not available.

This M13-based method of probe generation can be avoided by designing MLPA probe sets composed entirely of oligonucleotide probes that can be generated through chemical synthesis. Although several successful applications of fully synthetic MLPA probe sets have been reported (e.g., [24,25,26,27,28]), the vast majority of MLPA applications are still restricted to genes for which it is possible to use commercially available kits (MRC-Holland, http://www.mlpa.com).

Here we describe a protocol for the simple design and generation of MLPA assays that utilize exclusively synthetic probes. Critical modifications applied in our strategy are (1) a shortest probe length of 90 nt; (2) separation of subsequent probes by 3 and 4 nt for probes shorter and longer than 120 nt, respectively; (3) placing stuffer sequences into both 5’ and 3’ half-probes, making them of approximately equal length; and (4) restricting the longest probe/half-probe lengths to 200/100 nt, respectively. This leads to a capacity for analysis of 31 probes at once; longer oligonucleotide synthesis is also possible, expanding the capacity of this approach. A further increase of multiplexing capacity can be achieved by the use of two-color (or multiple-color) labeling on two distinct pairs of universal primers that enable a simultaneous CE analysis of two sets of MLPA products[24]. The strategy described here can be applied to any genomic region(s) of interest. We have used this strategy to generate over 10 different MLPA assays (examples are shown in Fig. 2). Published applications include the identification of large mutations in *TSC1*, *TSC2*[6], and *PKD1*[18] genes; analysis of loss of heterozygosity in cancer samples[23]; genotyping of several mouse transgenes[21]; and strand-specific expression analysis (Mykowska et al., submitted for publication).

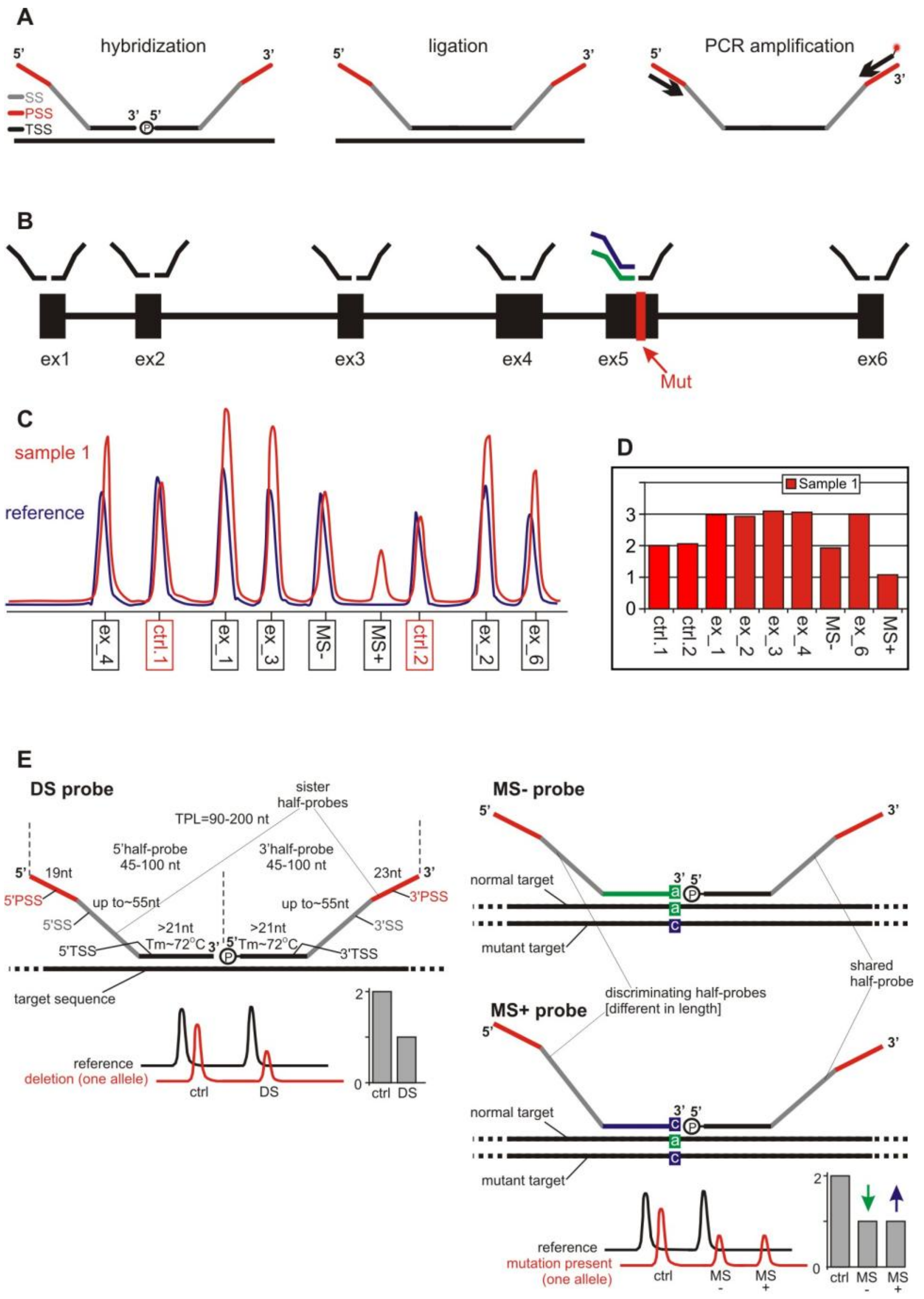

**FIGURE 1.** The principle of MLPA analysis for simultaneous identification of CNV and small mutations. (A) Three subsequent steps in the MLPA reaction (from left to right): hybridization of sister half-probes to the target sequence, ligation of correctly hybridized probes, and PCR amplification of ligated probes with universal primers. Primer-specific sequences (PSSs), stuffer sequences (SSs), and target-specific sequences (TSSs) are indicated in red, gray, and black, respectively. (B) Structure of a hypothetical model gene with the locations of MLPA probes (above). The probe located in exon 5 has two alternative 5' half-probes: one (MS-) specific for normal (green) and the other (MS+) specific for mutant (blue) sequence. The alternative 5' half-probes are different in length. (C) Overlapped hypothetical electropherograms of subject (red) and reference (blue) samples. Probe IDs are indicated below the electropherogram. (D) Bar graph showing relative copy number values calculated for each probe. Increased signal from all exonic probes (ex_1 to ex_6) indicates entire gene duplication. Relatively low signal from probe MS- located in exon 5 indicates the presence of a small mutation that is additionally confirmed by the appearance of a signal from the mutation-specific (MS+) probe. (E) Characteristics of the three types of MLPA probes; (left-hand side) copy number–sensitive (DS) probe, (above, right-hand side) small-size mutation–sensitive, negative (MS-) probe, and (below, right-hand side) small-size mutation–sensitive, positive (MS+) probe. In each upper panel, a schematic representation of an MLPA probe hybridized to its target sequence is shown. PSSs, SSs, and TSSs are indicated and marked as in panel A. TSSs specific for normal and mutant sequences are indicated in green and blue, respectively. In panels DS and MS (below), a schematic electropherogram of the analyzed (red line) and reference (black line) sample is shown. The results of copy number analysis presented in the form of a bar plot are shown below on the right-hand side.

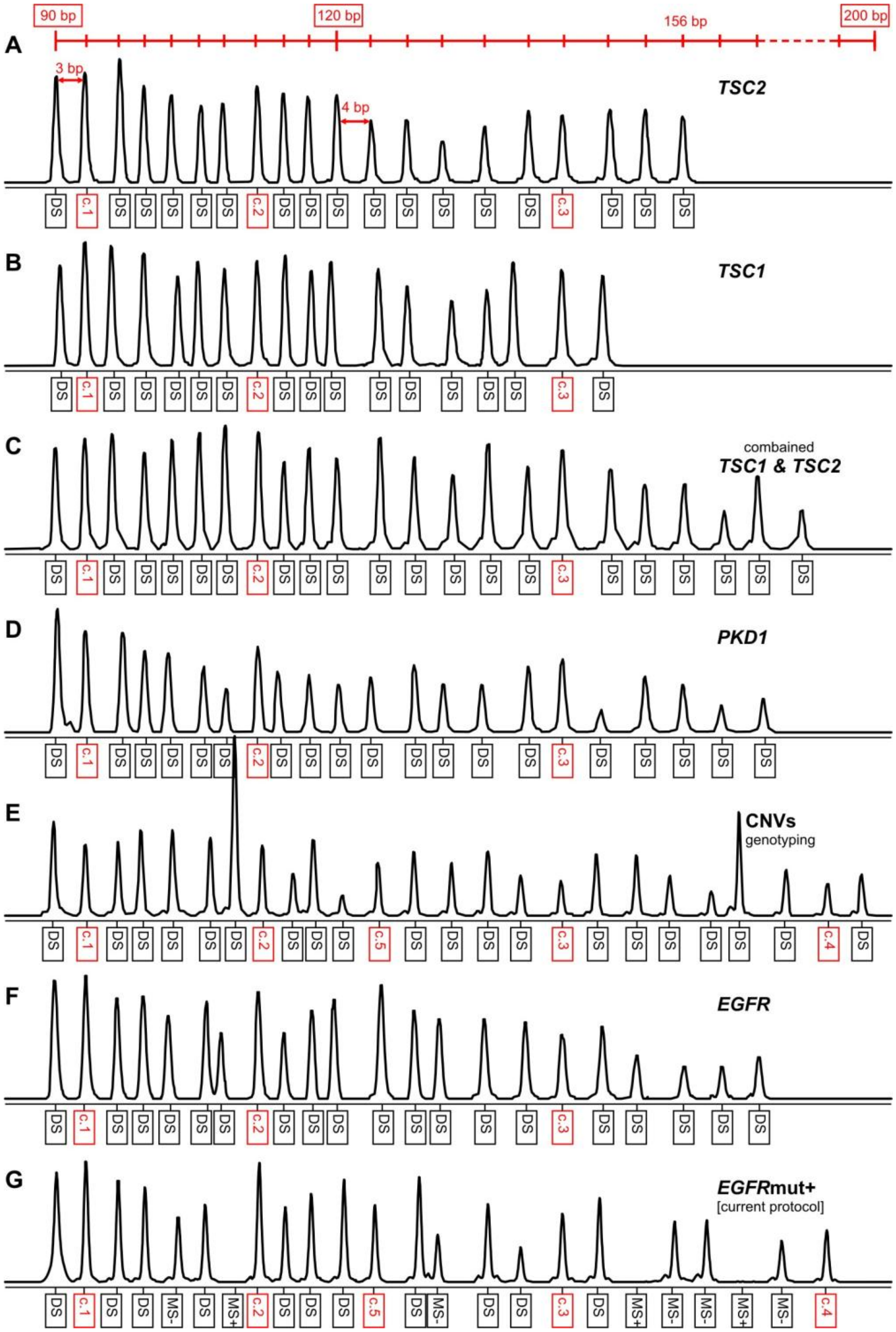

**FIGURE 2.** Examples of MLPA probe sets designed according to the described protocol. Electropherogram profiles representing a normal DNA sample analyzed with different MLPA probe sets (the signal of each probe [except panel E] represents two target sequence copies). (Top) Schematic representation of an MLPA probe set layout. Probe sets for large-mutation analysis in (A) *TSC2*, (B) *TSC1*, (C) both *TSC1* and *TSC2*, and (D) *PKD1*. (E) Probe set for genotyping several polymorphic CNVs at different sites in the genome. (F) Probe set for CNV analysis of *EGFR*. (G) Probe set for combined copy number and small-mutation analysis of *EGFR* (the assay described in this article). The types of the MLPA probes are indicated under the electropherograms. Control probes are indicated in red.

As an example, we present here the design of an MLPA probe set (assay) for the combined copy number and small-mutation analysis of the *EGFR* gene. *EGFR* is a well-known tumor proto-oncogene frequently mutated in various types of cancer. Oncogenic variants activating *EGFR* can be both copy number (*EGFR* amplification and vIII deletion) and small-size mutations (substitutions, in-frame deletions, and in-frame insertions)[29]. The status of *EGFR* mutations is an important factor modifying the effectiveness of tyrosine kinase inhibitor (TKI) treatment (reviewed in [30]). Lung cancers with certain *EGFR* mutations (e.g., L858R and exon 19 in-frame deletions) are sensitive to TKI treatment[31,32], whereas the occurrence of the secondary mutation T790M causes resistance to TKI[33,34].

The proposed MLPA setup allows for copy number or combined copy number and small-mutation analysis of up to ~30 genomic locations (probes) with a per-sample cost of ~$3 plus a starting cost (probe synthesis) of about $3000 (once synthesized, the number of probes obtained is sufficient for hundreds of thousands of analyses).

## MATERIALS

1. Reagents
   - A. MLPA reactions
     - (i) Genomic DNA sample: 20–50 ng/μl (3 μl per assay)
     - (ii) Probe mix: composed of self-designed synthetic probes. Synthesis parameters: synthesis scale, 100 nmol; purification: IE HPLC; modification, 5’ phosphorylation (only 3’ half-probes) (IDT-DNA)
     - (iii) MLPA reagent kit (includes all reagents except probe mix): SALSA MLPA Reagents (MRC-Holland EK1, EK5, EK20, or EK50)
     - (iv) Deionized water (resistance <18 MΩ cm)
   - B. Sample preparation and CE analysis
     - (i) HiDi formamide (Applied Biosystems Cat. No. 4311320)
     - (ii) CE polymer: ABI POP7 (Applied Biosystems)
     - (iii) DNA size standard: Gene Scan LIZ-600 (Applied Biosystems)
2. Equipment and consumables
   - A. 96-well plates: Certified Thin Wall 96 × 0.2 ml PCR Plates (Starlab)
   - B. PCR thermocycler: GeneAmp PCR System 9700 (Applied Biosystems) or PTC-200 Thermo Cycler (MJ Research)
   - C. Capillary electrophoresis: CE analysis can be performed on any standard multicapillary DNA analyzer (e.g., ABI-Prism 3130XL, 3100, 1700 [Applied Biosystems], CEQ-2000, 8000, 8800 [Beckman])

## PROCEDURE

### 1. General MLPA design

### A. Probe set layout

The MLPA assay can be composed of up to 31 probes, with a total probe length (TPL) ranging from 90 to 200 nt (half-probe length [HPL] ranging from 45 to 100 nt). The (EGFRmut+) MLPA probe set presented in this protocol was composed of 24 probes with TPL ranging from 90 to 172 nt. The difference between the lengths of the probes (spacing) was 3 and 4 nt for probes shorter and longer than 120 nt, respectively (Fig. 2).

COMMENT: The proposed spacing of the probe lengths ensures proper separation of PCR products during CE. Smaller differences in length can cause the adjacent peaks to overlap, making interpretation difficult. Larger spacing intervals can be used, but this reduces the capacity of an MLPA assay.

Most probes in the set are used to investigate CNV in the genomic region(s) of interest. Probes should be evenly distributed over the investigated region. If the region of interest contains a gene, probes can be preferentially located in exons.

COMMENT: The lengths of the probes do not have to correspond to the order of their genomic locations. Mixing up the lengths of the probes allows CNV to be distinguished from artifacts related to the size of the probes. True CNVs often affect probes that are located in adjacent positions in the genome, whereas length-dependent artifacts affect probes of similar lengths. The most common length-dependent artifact is a gradual increase or decrease of relative signal intensity corresponding to the probe length.

Each probe set should contain at least a few control probes (in the EGFRmut+ probe set, five control probes specific for locations in different chromosomes were used). The control probes should be chosen from outside the genomic region of interest, ideally from different chromosomes, and not subject to CNV in the general population. The Database of Genomic Variations (DGV - http://projects.tcag.ca/variation/) and other resources[11,12,35] can be used to avoid known CNV regions. Alternatively, the control probes proposed here (Supplementary Table 1) can be used. If an MLPA assay is intended to analyze somatic variation in cancer samples, the control probes should not be located within or close to known cancer-variable regions (e.g., proto-oncogenes and tumor suppressors)[36]. Recently published results of genome-wide somatic CNV analysis across numerous cancer samples[2] can be used to avoid regions highly variable in cancers.

### B. Probe layout

Each probe is composed of two sister half-probes (a 5' half-probe and a 3' half-probe) of equal length (Fig. 1). In the case of probes with an odd TPL, the length of sister half-probes can differ by 1 nt. Each half-probe consists of a target-specific sequence (TSS), a universal primer-specific sequence (PSS), and a stuffer sequence (SS) that allows the TPL to be modulated. The 3' half-probe is phosphorylated at its 5' end to enable ligation of sister half-probes. The 5' half-probe is composed of (from the 5' end): 5' PSS (19 nt), 5' SS (variable length), and 5' TSS (≥21 nt). The 3' half-probe is composed of (from the 5' end): 5' phosphate, 3' TSS (≥21 nt), 3' SS (variable length), and 3' PSS (23 nt) (Fig. 1E).

MLPA probe design depends on the purpose of the probe. The subsequent steps of the protocol describe the design of three types of MLPA probes that were used in the EGFRmut+ assay: (1) dosage (copy number) sensitive (DS); (2) small-size mutation sensitive, negative (MS-); and (3) small-size mutation sensitive, positive (MS+) probes (Fig. 1E).

## 2. Design of DS probes: general MLPA probe design

The basic MLPA probe is a DS probe (Fig. 1E). The signal intensity of a DS probe corresponds to the dosage (copy number) of the target sequence.

## A. Selection of TSSs

The TSSs specifically recognize analyzed sequences and are thus the most critical part of MLPA probes. The design of TSSs depends on the purpose of the probe.

(i) Select a genomic region or a gene of interest, retrieve genomic sequence, and paste it into a word-processing program (e.g., MS Word) (Supplementary File 1).
(ii) In the sequence of interest, mark exons or other sequences on which you are going to focus.
(iii) Label/mask the sequence of interest with (1) repeat/low-complexity regions, (2) positions of SNPs, (3) segmental duplication, and (4) polymorphic CNV regions.

COMMENT: There are many alternative resources that can be used for sequence selection (e.g., UCSC Genome Browser [UCSC GB]: http://genome.ucsc.edu/; Ensembl: http://www.ensembl.org; NCBI: http://www.ncbi.nlm.nih.gov; SNPper: http://snpper.chip.org) and labeling/masking (UCSC DB; DGV; RepeatMasker: http://www.repeatmasker.org/; dbSNP database: http://www.ncbi.nlm.nih.gov/snp/; HapMap: www.hapmap.org/).

(iv) [This step is a convenient alternative to steps (i–iii).] The sequence of interest marked with all the above-mentioned genetic features can be retrieved from UCSC GB in a few steps: (1) select the sequence of interest, (2) select “DNA” on the upper toolbar, (3) select “extend case/color options”, (4) fill in the “Extended DNA Case/Color Options” table as shown in Fig. 3, (5) press “submit”, and (6) copy the sequence to a word-processing program (Supplementary File 1).
(v) In the region for which the probe will be designed (e.g., exons), select candidate target sequences (about 100 nt long) free of polymorphisms and repetitive elements. Extremely high GC-content sequences should be avoided.
(vi) In the candidate target region, select directly adjacent 5’ and 3’ TSSs. Each TSS should be at least 21 nt long. Sister TSSs should be of similar lengths, and mononucleotide tracts of C or G (≥3 nt) should be avoided at or close to the ligation point. The melting temperature (Tm) of each TSS should be as close as possible to 71°C. To calculate the annealing temperature, use RaW-Probe v.0.15B. RaW-Probe is freely available at the MRC-Holland webpage (http://www.mlpa.com).

CAUTION: Avoid shortening the target sequence below 21 nt. If, due to high GC content, it is not possible to find any sequence with the optimal Tm value (71°C), it is better to select a sequence with a higher Tm rather than to shorten the sequence below 21 nt.

(vii) BLAST the selected TSSs against the appropriate reference sequence (here, the human genome) to verify that they are unique in the human genome. Use the algorithm BLASTN with the following parameters: no filtering, no repeat masking, and E (expectancy) = 1. We recommend using the BLAST program available at the NCBI webpage (http://blast.ncbi.nlm.nih.gov).
(viii) Mark selected TSSs in different colors (a different color for the 5’ TSS [e.g., yellow] and the 3’ TSS [e.g., green]) (Supplementary File 1) and paste them into the appropriate positions in the “Probe Set Assembly Table” (Supplementary Table 1).

COMMENT: The “Probe Set Assembly Table” serves as a tool for combining segments of half-probes into oligonucleotide sequences of the desired length. Each row represents one probe. Predefined probe lengths are indicated in the last column. Each row is divided into two

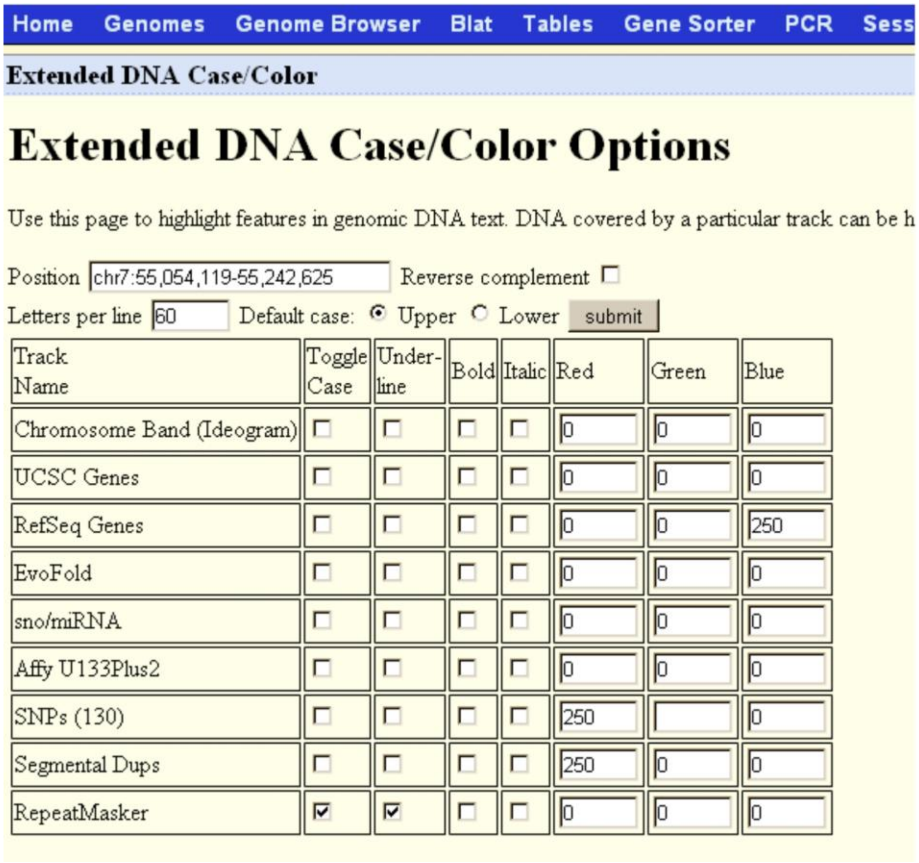

**FIGURE 3.** Screenshot of completed “Extended DNA Case/Color Options” table for labeling/masking DNA sequences retrieved from UCSC GB.

sections: a 5’ half-probe (yellow panels) and a 3’ half-probe (green panels). Each half-probe section includes columns with the sequences and lengths of the probe segments (from 5’): 5’ PSS, 5’ SS, 5’ TSS, 3’ TSS, 3’ SS, and 3’ PSS. The sequences and lengths of the PSSs as well as the sequences of the control probes can be pasted into the “Probe Set Assembly Table” prior to the start of a probe set designing project.

(ix) Use a strategy similar to that presented above (i–viii) to design control probes. Control probes should be located in genomic regions expected to be free of CNV in the intended experiments. Alternatively, control probes included in the EGFRmut+ set can be used as controls for any MLPA set. These control probes were already tested in several MLPA assays (Fig. 2).

## B. Addition of PSSs

The PSSs correspond to a pair of universal primers included in all commercially available MLPA reagent kits (MRC-Holland). They enable multiplex amplification of all MLPA probes.

(i) Paste the 5’ PSS (GGGTTCCCTAAGGGTTGGA) and 3’ PSS (TCTAGATTGGATCTTGCTGGCGC) into the appropriate positions of the “Probe Set Assembly Table” (Supplementary Table 1).

### C. Addition of SSs and assembly of half-probes

The SS is the sequence inserted between the PSS and the TSS to adjust both HPL and TPL.

(i) Using the following equations, calculate the length of the SS for each half-probe: length of 5' SS = predefined 5' HPL – (length of 5' PSS + length of 5' TSS); length of 3' SS = predefined 3' HPL – (length of 3' PSS + length of 3' TSS).

(ii) Paste the SSs of the appropriate length into the "Probe Set Assembly Table".

COMMENT: Although stuffers can be any sequence of appropriate length, we recommend using the appropriate fragments of the same universal SS in all probes. The universal SS used in all our MLPA sets is a 117-nt fragment of M13 sequence (AC# V00604) (Supplementary Table 1). This sequence was selected based on its GC content (49%), lack of substantial similarities to the human genome, and lack of any back-folding self-complementarities. The appropriate 5' and 3' SS fragments are generated from the 5' and 3' ends of the universal SS, respectively. Designing SSs in the way described above (and presented in Supplementary Table 1) substantially increases probe similarity in a way that extends well beyond the universal PSSs. This similarity significantly improves the uniformity of probe amplification and thus reduces amplicon-dependent signal variation. In all the designed MLPA sets, the relative signal intensity of most probes does not differ more than twofold (Fig. 2).

(iii) Combine the half-probe segments in the following order: 5' half-probe – 5' PSS, 5' SS, and 5' TSS; 3' half-probe – 3' TSS, 3'SS, and 3'PSS.

(iv) Again, BLAST all final probe sequences (combined 5' and 3' TSS) against the human genome to double check that no error was introduced during probe sequence assembly and handling. Use the BLAST parameters described above (step 2A vii). A correctly designed TSS should show (1) one perfectly matched sequence (the target) and (2) a lack of any other substantial similarities. Minor complementarities (e.g., <90% homology over 10 nt) to alternative genomic locations are acceptable.

## 3. Design of MS- probes

The MS- probe is a type of DS probe whose signal decreases in the presence of small mutations. Examples are shown in Fig. 4.

(i) Locate the position of the small mutation in the sequence of interest (Supplementary File 1; red-bold font).

(ii) Following the instructions described in step 2A, design a 5' and 3' TSS pair with a ligation position directly adjacent to the small mutation. The 3' end of the 5' TSS or the 5' end of the 3' TSS should overlap the mutation.

(iii) Add PSSs and SSs as described in step 2.

COMMENT: A single nucleotide mismatch at either the 5' or the 3' side of the ligation point will completely preclude ligation and subsequent amplification of the MS- probe. Note, however, that small-size mutations located outside of target sequences do not affect the probes signal and thus cannot be detected by MLPA.

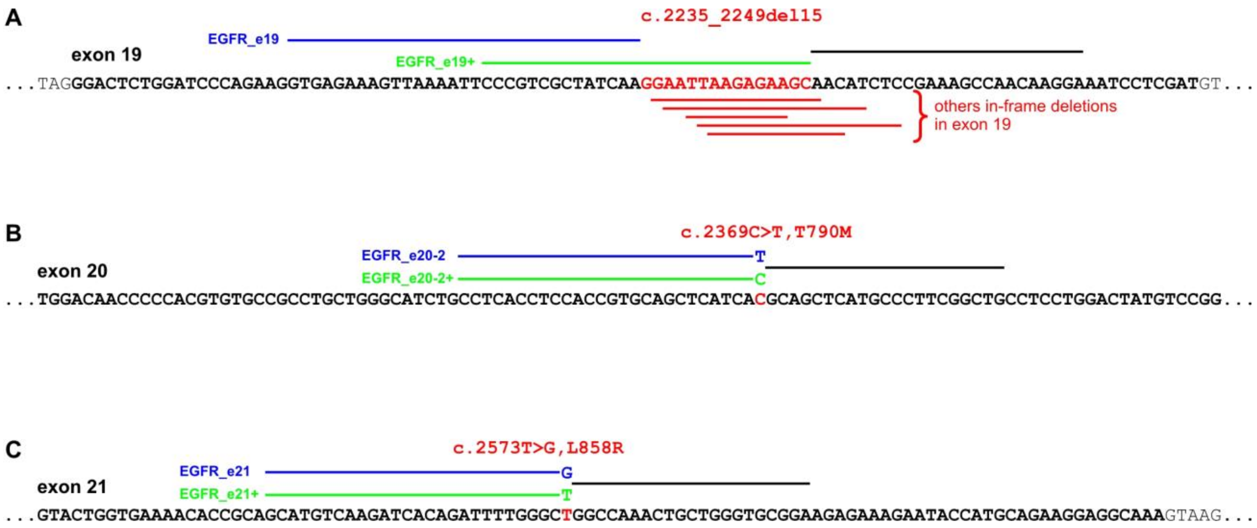

**FIGURE 4.** Pairs of target sequences for MS- and MS+ probes specific for (A) in-frame deletion c.2235_2249del15 in exon 19, (B) T790M in exon 20, and (C) L858R in exon 21. Black lines: 3' TSS shared by the MS- and MS+ probes; green and blue lines: 5' TSSs specific for the normal and mutant sequences, respectively.

## 4. Design of MS+ probes

The signal from MS+ probes appears only when a specific mutation is present. In the case of wild-type sequence, MS+ probes give no signal. Examples are shown in Fig. 4.

(i) Select a TSS for the MS- probe as described in step 3.
(ii) Replace either the 5' or the 3' TSS (depending on which one's end overlaps the mutation) of the MS- probe with the mutated TSS.
(iii) Add PSSs and SSs as described in step 2. One half-probe should be common for both MS- and MS+, and a second one should discriminate between the normal (MS-) and mutant (MS+) sequences (probes). Discriminating half-probes must be different in length (Fig. 1E).

## 5. Generation of half-probe oligonucleotides

(i) Order the oligonucleotide half-probes. There are many companies that provide oligonucleotide service suitable for generating MLPA probes. All half-probes used in our probe sets were synthesized by IDT (http://idtdna.com/) using the following parameters: synthesis scale, 100 nmol; purification IE HPLC; modification, 5' phosphorylation (3' half-probes only) (Supplementary Table 2).

CAUTION: To enable ligation of sister half-probes, all 3' half-probes must be phosphorylated at their 5' ends.

## 6. Preparation of the probe set

(i) Dilute all oligonucleotide half-probes with deionized water to a concentration of 20 μM (stock solutions).
(ii) Prepare a chart of a 96-well plate with individual positions designated for each half-probe oligonucleotide (Supplementary Table 2).

(iv) Aliquot 2 µl of each stock solution to the appropriate position in the 96-well plate. To each 2 µl of stock solution add 200 µl of deionized water and mix it well by carefully pipetting the mixture up and down (about 10 times). The concentration of oligonucleotides in the 96-well plate is 1 µM (working solutions).

(v) Mix 2 µl of each half-probe working solution in a 1.5-ml Eppendorf tube. Dilute the mixture with deionized water up to 400 µl (probe set mix).

## 7. MLPA reaction

(i) Use the prepared probe set mix as a standard "probemix" with SALSA MLPA reagents (MRC-Holland). Follow the standard MLPA protocol.

COMMENT: MLPA is a robust and easy-to-perform procedure. The MLPA protocol was described thoroughly in a seminal MLPA paper[16]. Additional information and troubleshooting can be found on the MRC-Holland webpage (www.mlpa.com). Therefore, detailed descriptions of the MLPA reactions and analysis are not part of this protocol.

## 8. CE of MLPA amplicons

The separation of MLPA amplicons can be performed on any standard multicolor capillary DNA analyzer (e.g., ABI-Prism 3100, 1700 [Applied Biosystems], CEQ-2000, 8000, 8800 [Beckman]). The general strategy for CE analysis and signal detection is similar with most commonly available apparatuses, but the detailed procedure differs from apparatus to apparatus and is described in detail in the appropriate manufacturers' manuals.

(i) Run the MLPA reaction under denaturing CE conditions following the detailed manufacturer's protocol. CE analysis of MLPA products is typically performed under the following conditions: sample dilution, 20–40× in deionized formamide; capillary length, ~42 cm; electroinjection voltage, 5 kV; electroinjection time, 5 sec; denaturing polymer, POP7 (Applied Bioscience); run temperature, 60°C; run voltage, 13 kV; electrophoresis time, ~20 min.

(ii) Using an appropriate program (e.g., GeneMapper [Applied Biosystems]), extract the probe signal intensity data (peak heights or peak areas).

COMMENT: We did not find any substantial difference using peak heights vs. peak areas as the probes' intensity representation; therefore, we routinely use only peak heights.

## 9. Analysis of MLPA results

(i) Divide the signals of all probes by the average signal of the control probes (signal normalization).

(ii) To calculate a copy number value for each probe, divide the normalized signal by the corresponding average normalized signal from a set of reference samples, and multiply by 2. We recommend the use of four reference samples.

COMMENT: The use of several reference samples in every experiment allows those reference results that show substantial deviation in relative probe signal intensity to be excluded, which reduces the effect of random signal variation occurring in individual (reference) samples.

(iii) The copy number values of all the analyzed probes can be visualized in the form of a bar graph, as shown in Figs. 1 and 5.

CAUTION: Most CNVs extend over long regions of the genome, and are often detected and validated by the simultaneous change in signal from multiple adjacent probes. Generally, for multiprobe CNVs, we recommend to assume that a signal-change threshold equals 2 standard deviations (SD) of a signal from unaffected probes[6,37,38]. In practice, multiprobe CNVs can be reliably detected by ≥20% increase (duplication) or decrease (deletion) of relative probe signals (assuming reasonably good-quality reactions with an unaffected probe signal SD of about 10%). This sensitivity allows not only for the detection of heterozygous mutations (50% signal change), but also for detection of heterozygous mosaic mutations affecting as little as 40% of cells from which the DNA has been extracted (20% signal change)[6,19,37]. However apparent CNV seen with a single probe may be artifactual or due to the presence of small mutations affecting the probe sequence. Therefore all single probe findings (including small-mutations detected by MS- probes) have to be validated by the use of alternative method.

**TIMING:** Designing a full set of MLPA probes (~25) takes 1–2 days (depending on the experimenter's skill and experience). Oligonucleotide dilution and probe mix preparation takes about 4 h.

## ANTICIPATED RESULTS

The protocol described in this paper was successfully used to design several different MLPA assays, including a test for combined copy number and small-mutation analysis of the *EGFR* gene (EGFRmut+). *EGFR* is composed of 28 exons spanning almost 200 kb of chromosome 7p11.2. Except for the extremely large intron 1 (over 100 kb), *EGFR* represents a typical human multiexon gene (Fig. 5A).

The reference sequence of *EGFR* extracted from UCSC GB (Supplementary File 1) shows exons (blue font), repetitive sequences (lowercase underlined font), and SNPs (red font). Additionally, the red-bold font indicates positions of the most common oncogenic *EGFR* mutations (labeled manually). Using the described protocol, a set of 24 probes was designed. The positions of the *EGFR* probes are indicated in the *EGFR* reference sequence (Supplementary File 1) (yellow and green highlight for 5' and 3' TSSs, respectively) and in Fig. 5A. The probes included five control probes (located on different chromosomes), 15 probes specific for *EGFR*, and four probes located in two other proto-oncogenes (two in *MET* [chr 7] and two in *ERBB2* [chr 17]). The *EGFR* gene probe set contained seven DS, five MS-, and three MS+ (Fig. 5). The mutations covered by the MS- probes accounted for over 90% of all oncogenic mutations occurring in the TK domain of *EGFR* (Fig. 5A). For the two most common *EGFR* mutations (L858R in exon 20 [~40%] and the most frequent in-frame deletion in exon 19, c.2235_2249del15 [~20%]) and for T790M in exon 20, probes specifically recognizing the mutant sequence (MS+) were also designed (Fig. 5).

The *EGFR* amplification that frequently occurs in different types of cancer resulted in increased relative signals from all DS and MS- probes. Also, other oncogenic rearrangements of *EGFR* could be detected as changes in DS and MS- probe signal intensity. An example of such a rearrangement is *EGFR* variant III (a large in-frame deletion including exons 2–8). Regardless of the copy number status of *EGFR*, the occurrence of specific small mutations resulted in a decrease of the relative signal of the corresponding MS- probe. This decrease was proportional to the number of *EGFR* copies in which the small mutation occurs. Additionally, in the case of three mutations (L858R, an in-frame deletion in exon 19 [c.2235_2249del15], and T790M), a signal from the corresponding MS+ probes should also occur.

The results of the EGFRmut+ MLPA analysis are shown in Fig. 5. The electropherograms shown in Fig. 5B represent one reference and two cancer samples (sample 1 and sample 2). The overlay of the reference and cancer sample electropherograms clearly shows an increase in *EGFR* probe signal in both

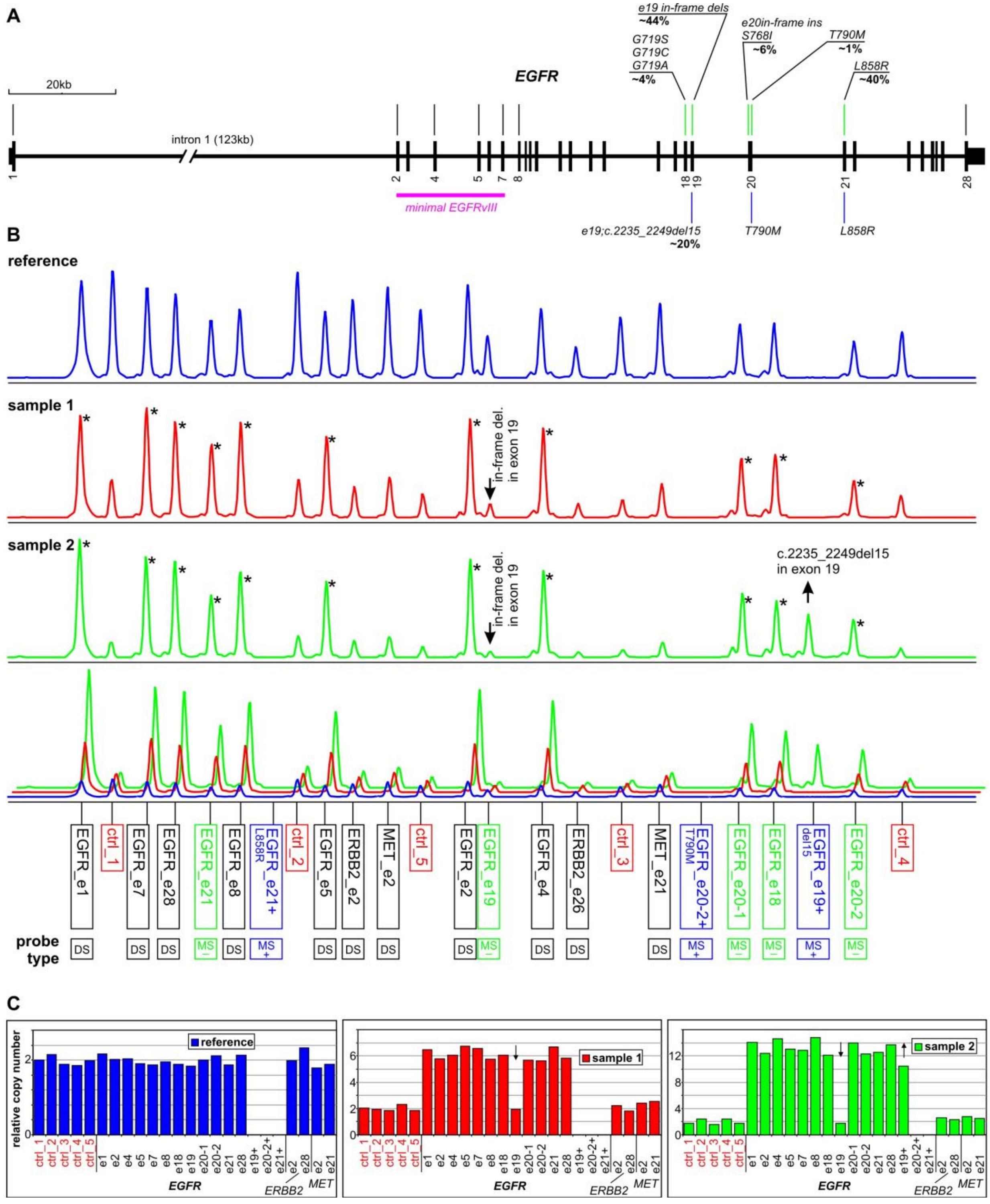

**FIGURE 5.** Representative results from the EGFRmut+ MLPA assay for combined copy number and small-mutation analysis of the *EGFR* gene. (A) Map of the *EGFR* gene with the positions of the EGFRmut+ probes indicated (vertical lines over and under the map). Black, green, and blue lines indicate the DS, MS-, and MS+ probes, respectively. The oncogenic small mutations (and their frequencies) covered by the MS- and MS+ probes are indicated over and under the corresponding probes, respectively. (B) Electropherograms represent (from the top) the reference sample, cancer sample 1, cancer sample 2, and an overlay of all three samples. Probe IDs and types are indicated under the electropherograms. Asterisks indicate amplified signals; arrowheads indicate reduced signal from MS- probe EGFR_e19 (specific for all in-frame deletions in exon 19) and increased signal of MS+ probe EGFR_e19+ (specific for deletion c.2235_2249del15). (C) Bar plots (corresponding to the electropherograms shown above [B]) represent the normalized copy number value (y-axis) of each probe (x-axis).

cancer samples (Fig. 5B and C), which corresponds to *EGFR* gene amplification up to six and 12 copies in samples 1 and 2, respectively. In both analyzed cancer samples, a lower signal from the EGFR_e19- probe is also clearly visible. The lower signal of this probe indicated the presence of one of the in-frame deletions in exon 19. Additionally, the signal of the EGFR_e19+ probe that appears in sample 2 and is clearly absent in the reference and sample 1 indicates that the in-frame deletion that occurred in sample 2 was c.2235_2249del15, which is the most common in-frame deletion in exon 19 and the second most common mutation in *EGFR*.

The protocol proposed here can be easily used to design an MLPA probe set for copy number analysis, or for combined copy number and small-mutation analysis of any region of interest in any genome. This strategy for parallel copy number and small-mutation analysis can be used to prescreen disease-related genes for large mutations and the most common recurrent small mutations.

## ACKNOWLEDGMENTS

This work was supported by the Ministry of Science and Higher Education, Grant No. N N302-278937 (PK and MM); Uniting Against Lung Cancer grant (DJK and K-KW); and Dana-Farber-Harvard Cancer Center Lung Cancer Specialized Program of Research Excellence (SPORE) grants P50 CA090578 (DJK, K-KW), U01 CA141576 (K-KW), R01 AG2400401 (K-KW), R01 CA122794 (K-KW), R01 CA140594 (K-KW), and 1RC2CA147940-01 (K-KW).
